# Classification of 27 *Corynebacterium kroppenstedtii*-Like Isolates Associated with Mastitis in China and Descriptions of *C. parakroppenstedtii* sp. nov. and *C. pseudokroppenstedtii* sp. nov

**DOI:** 10.1128/spectrum.01372-21

**Published:** 2022-03-15

**Authors:** Qiang Luo, Qianming Chen, Junhui Feng, Tianqi Zhang, Li Luo, Cha Chen, Xiaoyan Liu, Ning Xu, Pinghua Qu

**Affiliations:** a Department of Clinical Laboratory, the Second Affiliated Hospital of Guangzhou University of Chinese Medicine, Guangdong Provincial Hospital of Traditional Chinese Medicine, Guangzhou, Guangdong, China; b The Second Clinical College, Guangzhou University of Chinese Medicine, Guangzhou, Guangdong, China; c Department of Breast Centre, the Second Affiliated Hospital of Guangzhou University of Chinese Medicine, Guangdong Provincial Hospital of Traditional Chinese Medicine, Guangzhou, Guangdong, China; Montefiore Medical Center and Albert Einstein College of Medicine

**Keywords:** mastitis, breast pathogens, *Corynebacterium*, taxonomy, antimicrobial susceptibility

## Abstract

*Corynebacterium*, particularly Corynebacterium kroppenstedtii, has been increasingly recognized as an important pathogen causing mastitis. However, no clear taxonomic, microbiological, or clinical identification for C. kroppenstedtii-related *Corynebacterium* species is recognized. During the investigation of isolates cultured from female patients with mastitis, 27 lipophilic *C. kroppenstedtii*-like isolates were obtained from clinical breast specimens from 2017 to 2019 in Guangzhou, China. These isolates were identified by phenotypic characterization, matrix-assisted laser desorption ionization-time of flight mass spectrometry (MALDI-TOF MS), partial sequencing of the 16S rRNA, *rpoB*, and *fusA* genes, and whole-genome sequencing methods. By phylogenetic analyses, two major clusters were identified that were closely related to *C. kroppenstedtii* DSM 44385^T^. Comparative genome analyses suggested that these isolates formed two distinct genospecies within the genus *Corynebacterium*. The digital DNA–DNA hybridization (dDDH) values for the two genospecies were 45.5 to 47.8% between them and 47.4 to 47.7% and 49.9% to *C. kroppenstedtii* DSM 44385^T^, respectively. Based on these results, it can be concluded that these isolates need to be recognized as two new species of the genus *Corynebacterium*, for which we proposed the names Corynebacterium parakroppenstedtii sp. nov. and Corynebacterium pseudokroppenstedtii sp. nov. The type strain for the novel species *Corynebacterium parakroppenstedtii* is MC-26^T^ (NBRC 115146^T^; CCTCC AB 2020210^T^), and that for *Corynebacterium pseudokroppenstedtii* is MC-17X^T^ (NBRC 115143^T^; CCTCC AB 2020199^T^).

**IMPORTANCE** In this study, we characterized two novel species that were closely related to but hard to distinguish from *C*. *kroppenstedtii* by routine identification methods used in clinical laboratories. Since all 27 *C. kroppenstedtii*-like isolates were obtained from breast specimens of female patients with mastitis, they may be potential pathogens causing mastitis. We hope to perform further epidemiological investigation of these strains and explore their role in mastitis.

## INTRODUCTION

The genus *Corynebacterium* represents a large group of Gram-positive, non-spore-forming, rod-shaped bacteria within the family *Corynebacteriaceae* of the order *Corynebacteriales* (1). It comprises more than 130 species with validly described names and has been detected in various habitats, such as soil, food, animals, humans, and plant surfaces. Except for some particular species that are well-established pathogens of humans and animals, *Corynebacterium* spp. have often been assigned as opportunistic pathogens. They are common components of the skin microbiome and generally have been dismissed as contaminants when recovered from clinical specimens, although they have been increasingly recognized to be associated with clinical symptoms (2–4).

Several *Corynebacterium* species, Corynebacterium kroppenstedtii in particular, have been reported to be associated with mastitis, which is a chronic inflammatory disease of unknown etiology in parous women of reproductive age (5). C. kroppenstedtii which lacks the typical mycolic acids of the cell envelope was first documented in 1998 from a human sputum specimen (6). It has occasionally been associated with human infection, mainly breast abscesses and granulomatous mastitis (7–9). Although the virulence of *C. kroppenstedtii* in mastitis is not fully understood, it is often isolated alone and early in disease progression, suggesting its pathogenic role (9, 10). In recent years, increasing *C. kroppenstedtii* infection has been reported by newer techniques, such as matrix-assisted laser desorption ionization-time of flight mass spectrometry (MALDI-TOF MS) and 16S rRNA gene sequencing (7, 11–15).

In this study, we describe the characterization of 27 *C. kroppenstedtii*-like isolates obtained from female patients with mastitis at our hospital from 2017 to 2019 and identify them as two novel species of the genus *Corynebacterium*.

## RESULTS

### Clinical information of *C. kroppenstedtii*-like isolates.

Twenty-seven *C. kroppenstedtii*-like isolates were obtained from breast specimens of 27 different female patients with mastitis. Most isolates exhibited pure growth like that of *C. kroppenstedtii*, and some other isolates exhibited mixed growth with coagulase-negative Staphylococcus and other *Corynebacterium* spp. Further histological analyses were available for 13 patients. Granulomatous mastitis was observed in 12 patients, and suppurative mastitis was observed in 1 patient. The ages of the patients were in the range of 26 to 47 years (mean 34.3 years). Basic information of these 27 cases of *C. kroppenstedtii*-like infection is presented in Table 1.

**TABLE 1:** Data of 27 cases of *C. kroppenstedtii*-like isolates from Chinese clinical breast specimens

Strain no.	Isolation date	Age (yr)/sex^*a*^	Specimen source	Diagnosis^*b*^	Comorbidity	Treatment^*c*^	Prognosis
*C. kroppenstedtii*-like group I							
MC-01	2017	26/F	Pus, tissue	GLM	Hyperprolactinemia	Surgery, TCM, dexamethasone, bromocriptine	Recovery
MC-04	2017	36/F	Pus, tissue	SM	Unknown	Surgery, antibiotic: rifampicin, isoniazid, ethambutol, bromocriptine	Recovery
MC-05	2017	35/F	Pus, tissue	M	Unknown	Surgery, TCM, bromocriptine	Recovery
MC-06	2017	32/F	Pus, tissue	M	Pulmonary Tuberculosis	TCM, bromocriptine	Transfer to chest hospital
MC-08	2017	42/F	Pus, tissue	M	HBV carrier	Surgery, TCM, dexamethasone, bromocriptine	Recovery
MC-09	2017	42/F	Pus, tissue	GLM	Thalassemia	Surgery, TCM, bromocriptine	Recovery
MC-10	2017	33/F	Pus, tissue	M	Unknown	Surgery, TCM, bromocriptine	Recovery
MC-11	2017	28/F	Pus, tissue	GLM	Pituitary adenoma	Surgery, TCM, bromocriptine	Recovery
MC-12	2018	35/F	Pus	M	Unknown	Surgery, TCM, bromocriptine	Recovery
MC-13	2018	34/F	Pus, tissue	GLM	Unknown	Surgery, TCM, bromocriptine	Recovery
MC-15	2018	31/F	Pus, tissue	GLM	HBV carrier	Surgery, TCM, bromocriptine	Recovery
MC-16	2018	27/F	Pus	M	Unknown	Surgery, TCM, bromocriptine	Recovery
MC-19	2018	35/F	Secretion	M	Unknown	Surgery, TCM, bromocriptine	Recovery
MC-20	2018	35/F	Pus	M	Unknown	Surgery, TCM, bromocriptine	Recovery
MC-21	2018	30/F	Pus, tissue	GLM	Unknown	Surgery, TCM, bromocriptine	Recovery
MC-22	2018	36/F	Pus, tissue	M	Unknown	Surgery, TCM, bromocriptine	Recovery
MC-23	2018	33/F	Secretion	M	Unknown	Surgery, TCM, bromocriptine	Recovery
MC-24	2019	37/F	Pus, tissue	GLM	Unknown	Surgery, TCM, bromocriptine	Recovery
MC-25	2019	31/F	Pus, tissue	GLM	HBV carrier	Surgery, TCM, bromocriptine	Recovery
MC-26	2019	47/F	Secretion	M	Unknown	Surgery, TCM, bromocriptine	Recovery
MC-27	2019	47/F	Pus	M	Unknown	Surgery, TCM, bromocriptine	Recovery
MC-28	2019	38/F	Pus, tissue	GLM	Diabetes mellitus	Surgery, TCM, bromocriptine	Self-discharged
MC-29	2019	31/F	Puncture fluid	M	Unknown	Surgery, TCM, bromocriptine	Recovery
*C*. *kroppenstedtii*-like group II							
MC-02	2017	42/F	Secretion	GLM	Hyperprolactinemia	Surgery, TCM, bromocriptine	Recovery
MC-03	2017	26/F	Pus, tissue	M	Unknown	Surgery, TCM, bromocriptine	Recovery
MC-07	2017	31/F	Pus, tissue	GLM	Unknown	Surgery, TCM, bromocriptine	Recovery
MC-17X	2018	27/F	Pus	M	Unknown	Surgery, TCM, bromocriptine	Self-discharged

a F, female.

b GLM, granulomatous lobular mastitis; SM, suppurative mastitis; M, mastitis.

c Surgery included abscess incision drainage and debridement. TCM, traditional Chinese medicine.

### Phenotypic testing and MALDI-TOF MS.

All isolates grew on Columbia blood agar plates as grayish, smooth, circular, convex, and nonhemolytic colonies of less than 1 mm in diameter after 72 h of incubation at 35°C in the presence of 5% CO_2_ atmosphere. On microscopic examination, cells were Gram-positive, rod-shaped, nonmotile, and non-spore-forming with typical coryneform morphology. All isolates were lipophilic, and the growth was enhanced on brain heart infusion broth and Columbia blood agar supplemented with 1% Tween 80. In general, the cellular and colony morphologies of these 27 isolates were similar to those of *C. kroppenstedtii* DSM 44385^T^.

All the 27 isolates, along with the type strain *C. kroppenstedtii* DSM 44385^T^, were found to be catalase and pyrazinamidase positive but nitrate and urease negative. Exceptions were the case of isolate MC-28, which was pyrazinamidase negative and isolate MC-11 urease positive. They produced acid from glucose but not from sucrose, ribose, or xylose (exception: isolate MC-27 could not produce acid from glucose). Results of API Coryne assay indicated that 25 of the isolates and the type strain showed 87.5 to 99.5% identity to Corynebacterium argentoratense, while 2 isolates were similar to Corynebacterium urealyticum with 60.5% and 8.8% identity, respectively (*C. kroppenstedtii* is not included in the API database). The characteristics produced by API Coryne strips are shown in Table 2, and the comparative analyses with *C. kroppenstedtii* DSM 44385^T^ are shown in Table 3.

**TABLE 2:** Polyphasic identification results of *C. kroppenstedtii*-like isolates

Strain no.	API coryne^*a*^		Strain identified by MALDTOF-MS^*b*^ in database:		Partial 16S rRNA gene^*c*^		Partial *rpoB* gene^*c*^		Partial *fusA* gene^*c*^		Genome GenBank accession no.
	Profile no. obtained	Significant taxon (% ID, T^*d*^)	Biotyper database (score value)	Vitek database (confidence value)	Similarity	GenBank accession no.	Similarity	GenBank accession no.	Similarity	GenBank accession no.	
TS	2040104	Car (87.5, 0.49)	Ckr CCUG 44504 (2.39)	Ckr 99.9%		NR_074408.1		AY492274.1		CP001620.1	CP001620.1
*C. kroppenstedtii*-like group I											
MC-01	2040104	Car (87.5, 0.49)	Ckr CCUG 49276 (1.643)	Ckr 99.9%	99.6%	MW819649	96.2%	MZ031080	97.7%	MZ031107	JAKJKX000000000
MC-04	2040104	Car (87.5, 0.49)	Gcr DSM 15881^T^ (1.583)	Ckr 99.9%	99.6%	MW819652	96.2%	MZ031083	97.7%	MZ031110	JAKJKY000000000
MC-05	2000104	Car (99.5, 0.99)	Ckr DSM 44385^T^ (1.579)	Ckr 99.9%	99.6%	MW819653	96.2%	MZ031084	97.7%	MZ031111	JAKJKZ000000000
MC-06	2000104	Car (99.5, 0.99)	Ckr CCUG 44504 (2.077)	Ckr 99.9%	99.6%	MW819654	96.2%	MZ031085	97.7%	MZ031112	JAKJLA000000000
MC-08	2000104	Car (99.5, 0.99)	Ckr CCUG 49276 (1.384)	Ckr 99.9%	99.6%	MW819656	96.2%	MZ031087	97.7%	MZ031114	JAKLTI000000000
MC-09	2000104	Car (99.5, 0.99)	Ckr CCUG 44504 (1.401)	Ckr 99.9%	99.6%	MW819657	96.2%	MZ031088	97.7%	MZ031115	JAKKNX000000000
MC-10	2000104	Car (99.5, 0.99)	Ckr DSM 44385^T^ (1.687)	Ckr 99.9%	99.6%	MW819658	96.2%	MZ031089	97.7%	MZ031116	JAKKNY000000000
MC-11	2041104	Cur (60.5, 0.27)	Ckr CCUG 44504 (1.892)	Ckr 99.9%	99.9%	MW819659	96.2%	MZ031090	97.6%	MZ031117	JAFFSY000000000
MC-12	2040104	Car (87.5, 0.49)	Ckr DSM 44385^T^ (1.465)	Ckr 99.9%	99.6%	MW819660	96.2%	MZ031091	97.7%	MZ031118	JAKJKU000000000
MC-13	2040104	Car (87.5, 0.49)	Ckr CCUG 44504 (1.709)	Ckr 99.9%	99.6%	MW819661	96.2%	MZ031092	97.7%	MZ031119	JAKKFA000000000
MC-15	2040104	Car (87.5, 0.49)	Ckr CCUG 44504 (1.828)	Ckr 99.9%	99.6%	MW819662	96.2%	MZ031093	97.7%	MZ031120	JAKLTJ000000000
MC-16	2040104	Car (87.5, 0.49)	Ckr CCUG 44504 (1.635)	Ckr 99.9%	99.6%	MW819663	96.2%	MZ031094	97.7%	MZ031121	JAKJKV000000000
MC-19	2000104	Car (99.5, 0.99)	Ckr DSM 44385^T^ (1.602)	Ckr 99.9%	99.6%	MW819665	96.2%	MZ031096	97.7%	MZ031123	JAKJKW000000000
MC-20	2000104	Car (99.5, 0.99)	Ckr CCUG 44504 (1.924)	Ckr 99.9%	99.6%	MW819666	96.2%	MZ031097	97.7%	MZ031124	JAKJKP000000000
MC-21	2040104	Car (87.5, 0.49)	Ckr CCUG 44504 (1.927)	Ckr 99.9%	99.6%	MW819667	96.2%	MZ031098	97.7%	MZ031125	JAKJKQ000000000
MC-22	2040104	Car (87.5, 0.49)	Ckr DSM 44385^T^ (1.419)	Ckr 99.9%	99.6%	MW819668	96.2%	MZ031099	97.7%	MZ031126	JAKJKR000000000
MC-23	2040104	Car (87.5, 0.49)	Ckr CCUG 44504 (1.622)	Ckr 99.9%	99.6%	MW819669	96.2%	MZ031100	97.7%	MZ031127	JAKJKS000000000
MC-24	2000104	Car (99.5, 0.99)	Ckr DSM 44385^T^ (1.441)	Ckr 99.9%	99.7%	MW819670	96.2%	MZ031101	97.7%	MZ031128	JAGSNZ000000000
MC-25	2000104	Car (99.5, 0.99)	Ckr CCUG 61180 (1.467)	Ckr 99.9%	99.6%	MW819671	96.2%	MZ031102	97.7%	MZ031129	JAKKOA000000000
MC-26	2000104	Car (99.5, 0.99)	Ckr DSM 44385^T^ (1.423)	Ckr 99.9%	99.6%	MW819672	96.2%	MZ031103	97.7%	MZ031130	JAGSOA000000000
MC-27	2040004	Cur (8.8, 0.44)	Ckr CCUG 44504 (1.966)	Ckr 99.9%	99.6%	MW819673	96.2%	MZ031104	97.7%	MZ031131	JAKJKT000000000
MC-28	0000104	Car (90.3, 0.65)	Ckr DSM 44385^T^ (1.597)	Ckr 99.9%	99.6%	MW819674	98.1%	MZ031105	97.4%	MZ031132	JAGSNY000000000
MC-29	2040104	Car (87.5, 0.49)	Ckr CCUG 49276 (1.603)	Ckr 99.9%	99.6%	MW819675	96.2%	MZ031106	97.6%	MZ031133	JAKJKO000000000
*C. kroppenstedtii*-like group II											
MC-02	2040104	Car (87.5, 0.49)	Ckr CCUG 44504 (2.276)	Ckr 99.9%	99.9%	MW819650	96.2%	MZ031081	97.3%	MZ031108	JAKKNZ000000000
MC-03	2040104	Car (87.5, 0.49)	Ckr CCUG 44504 (2.029)	Ckr 99.9%	99.9%	MW819651	96.2%	MZ031082	97.3%	MZ031109	JAKLTK000000000
MC-07	2040104	Car (87.5, 0.49)	Ckr CCUG 44504 (2.316)	Ckr 99.9%	99.9%	MW819655	96.2%	MZ031086	97.3%	MZ031113	JAKJLB000000000
MC-17X	2040104	Car (87.5, 0.49)	Ckr CCUG 44504 (2.295)	Ckr 99.9%	99.9%	MW819664	96.2%	MZ031095	97.3%	MZ031122	JAEUWU000000000

a Car, *C. argentoratense*; Cur, *C. urealyticum*.

b Ckr, *C. kroppenstedtii*; Gcr, Glutamicibacter creatinolyticus.

c Similarities of partial 16S rRNA, *rpoB*, and *fusA* genes are presented with respect to the genome of *C. kroppenstedtii* DSM 44385^T^.

d TS, the type strain *C. kroppenstedtii* DSM 44385^T^; T, typicity index.

**TABLE 3:** Phenotypic characteristics of the two groups of *C. kroppenstedtii*-like isolates and the type strain

Characteristics	Result for strains^*a*^		
	*C. kroppenstedtii*-like group I (*n* = 23)	*C. kroppenstedtii*-like group II (*n* = 4)	*C. kroppenstedtii* DSM 44385^T^
Lipophilism	100	100	+
Catalase	100	100	+
Nitrate reduction	0	0	−
Hydrolysis of aesculin	56	100	+
Hydrolysis of urea	4	0	−
Enzyme activity			
Pyrazinamidase	95	100	+
Alkaline phosphatase	0	0	−
Acid production from			
Glucose	95	100	+
Sucrose	0	0	−
Ribose	0	0	−
Xylose	0	0	−

a Numbers represent percentages of positive results. +, positive reaction; −, negative reaction.

By the Vitek MS system (V2.0; bioMérieux, France), all 27 isolates were identified to *C. kroppenstedtii* with the confidence value of 99.9%. By the Bruker Biotyper system (BDAL Library; Bruker Daltonics, Germany), only 5 isolates (MC-02, MC-03, MC-06, MC-07, MC-17X) had good identification to species level (scores of ≥2.0) for *C. kroppenstedtii* CCUG 44504 as the “rank 1” identification, and another 6 produced scores of ≥1.7 for *C. kroppenstedtii* CCUG 44504 as the rank 1 identification. The remainders had values below the accepted score for reliable identification (scores of <1.7). The MALDI-TOF MS results are shown in Table 2.

**FIG 1:**
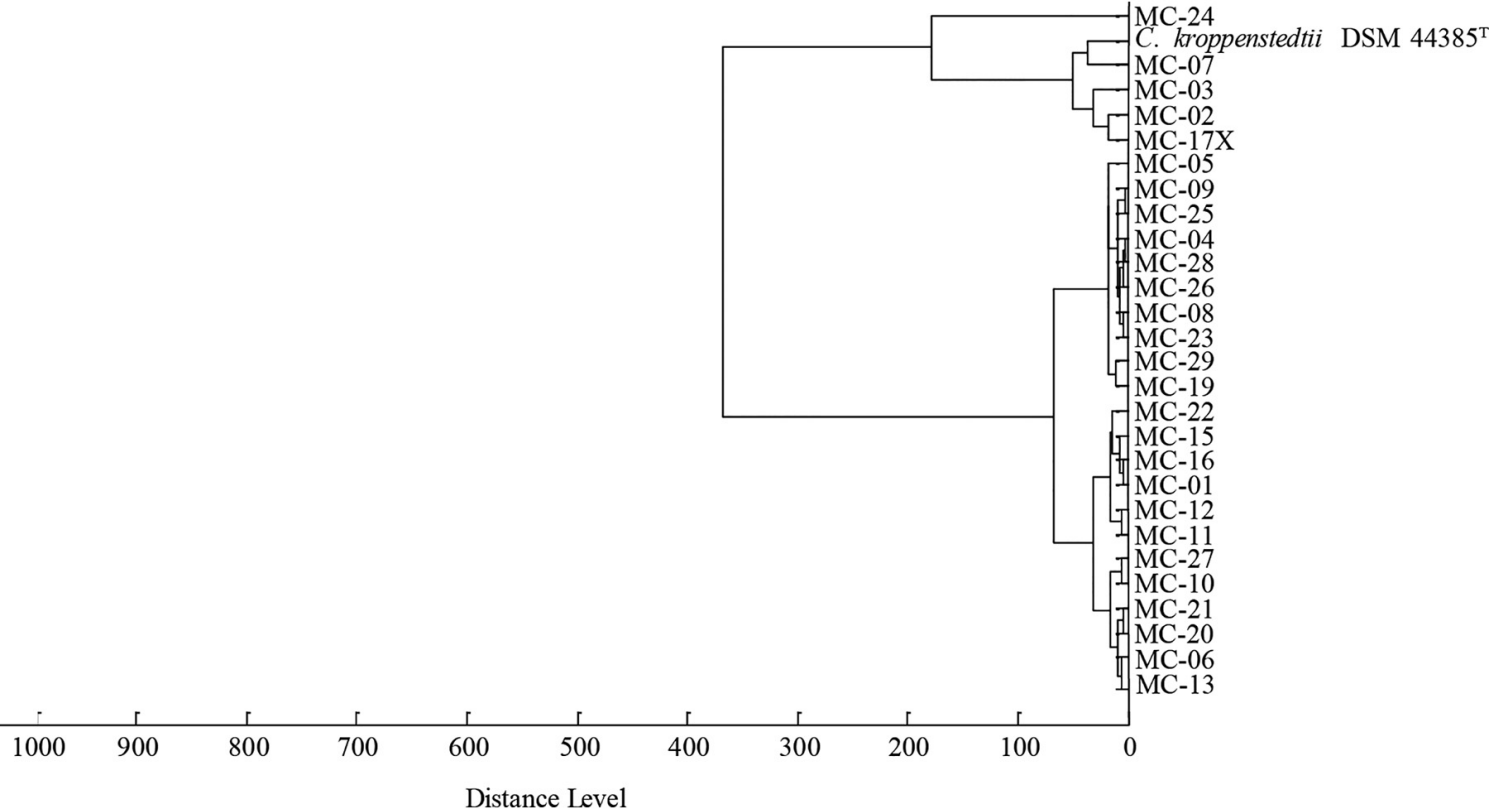
Dendrogram revealed by MALDI-TOF MS (Bruker Biotyper system) showing the relationship of 27 *C. kroppenstedtii*-like isolates and the type strain.

On the basis of the data revealed by MALDI-TOF MS (Bruker Biotyper), 4 isolates (MC-02, MC-03, MC-07, MC-17X) clustered with the type strain *C. kroppenstedtii* DSM 44385^T^, 22 isolates (MC-01, MC-04, MC-05, MC-06, MC-08, MC-09, MC-10, MC-11, MC-12, MC-13, MC-15, MC-16, MC-19, MC-20, MC-21, MC-22, MC-23, MC-25, MC-26, MC-27, MC-28, MC-29) constituted a coherent and distinct cluster separate from the type strain, and the remaining one isolate (MC-24) showed a unique position (Fig. 1).

### Genotypic identification.

To further identify the taxonomic position, the clinical isolates were subjected to partial sequencing of the 16S rRNA gene (about 1,350 bp), *rpoB* gene (about 420 bp), and *fusA* gene (about 990 bp) and comparative sequence analysis (Table 2), and clustering analysis of the clinical isolates along with the type strain were done.

**FIG 2:**
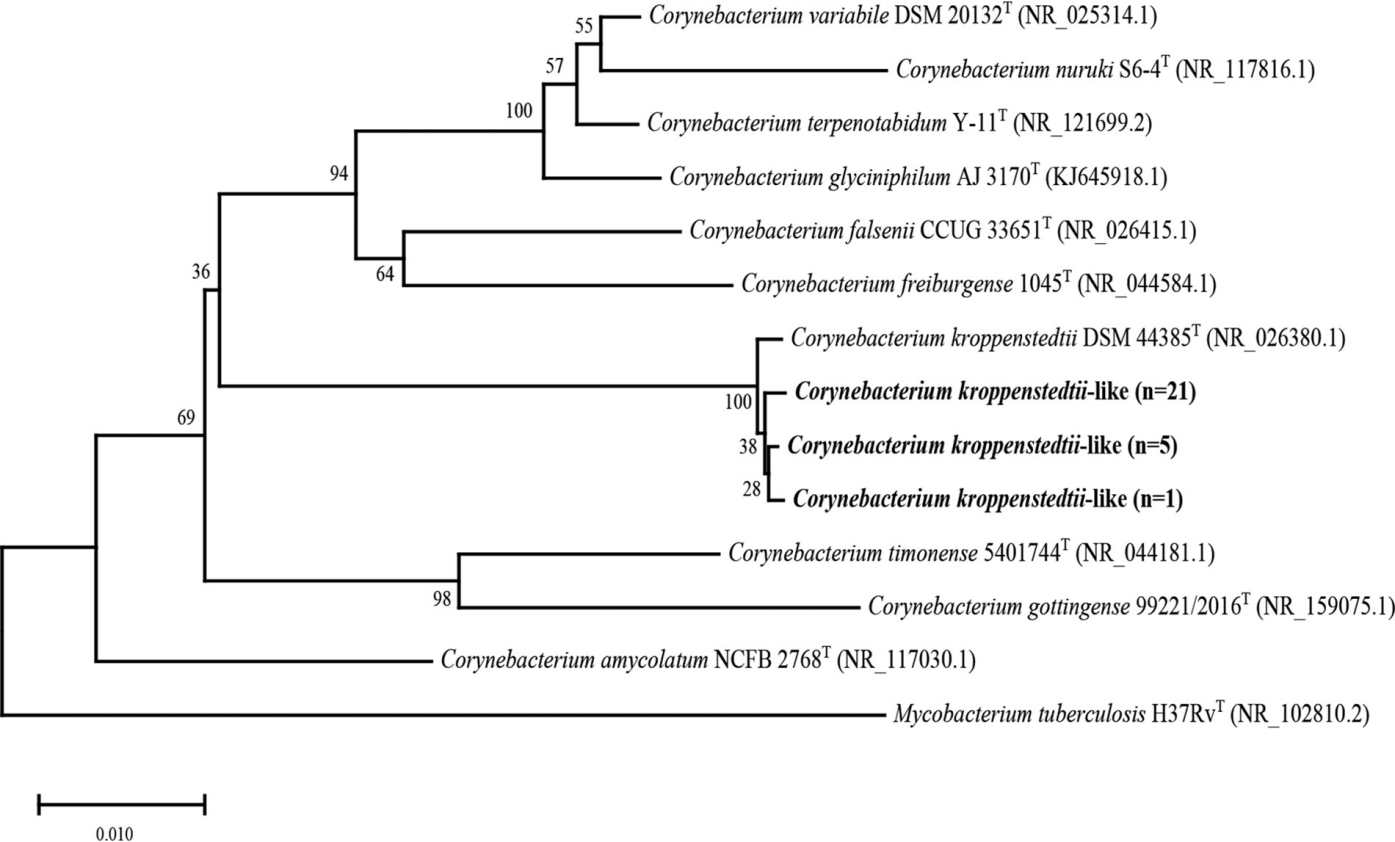
Neighbor-joining tree based on partial 16S rRNA gene showing the phylogenetic relationship of 27 *C. kroppenstedtii*-like isolates and the most closely related species in the genus *Corynebacterium*. Bootstrap values based on 1,000 calculations are shown. The scale bar depicts 0.010 substitutions per nucleotide position.

Based on phylogenetic analyses of the partial 16S rRNA gene, all the 27 isolates were closely related to *C. kroppenstedtii* DSM 44385^T^ (Fig. 2), exhibiting 99.6 to 99.9% similarities. The *rpoB* and *fusA* genes of the 27 isolates showed sequences identities of 96.2 to 98.1% and 97.3 to 97.7% with *C. kroppenstedtii* DSM 44385^T^, respectively.

**FIG 3:**
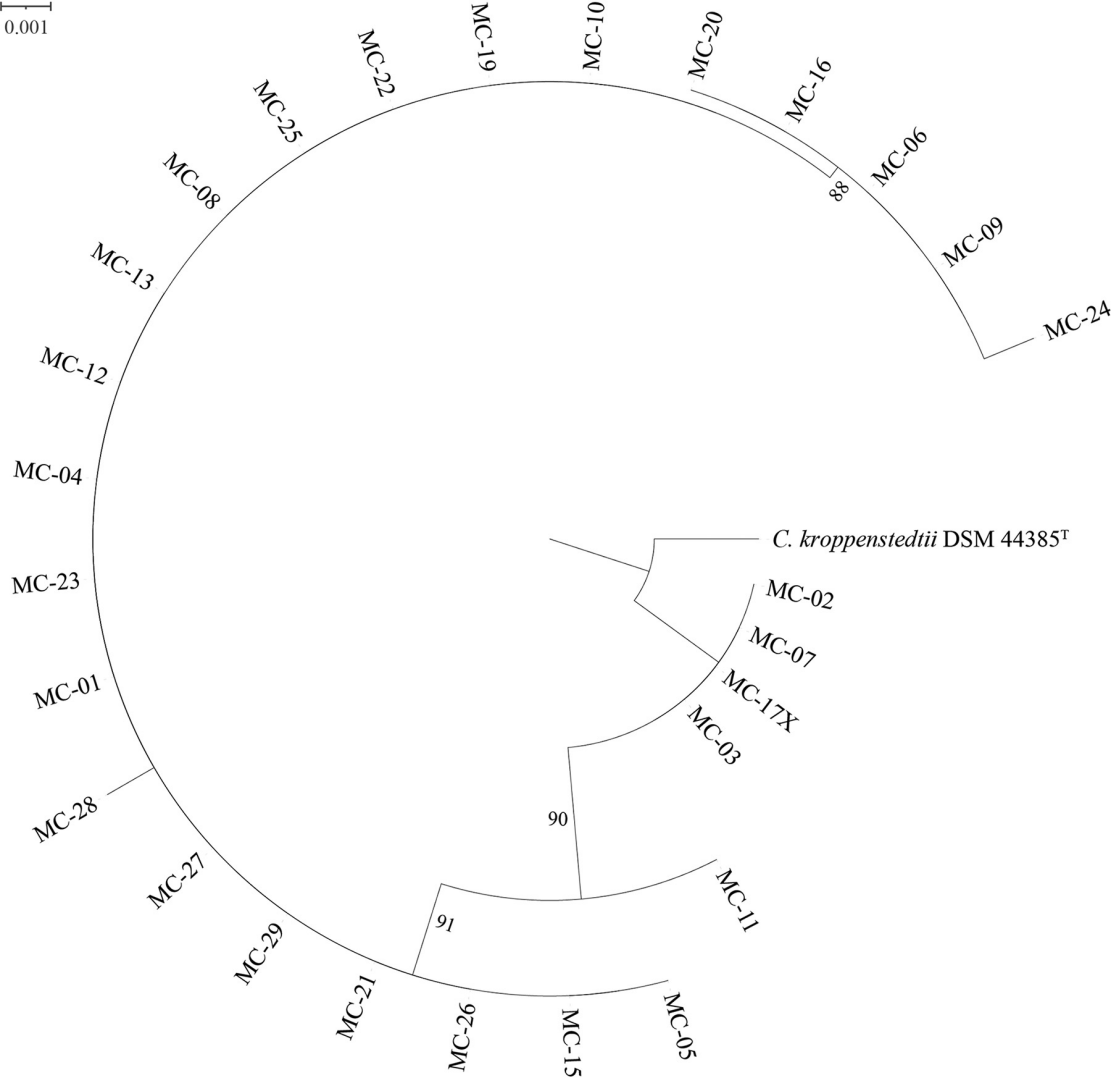
Protein-concatemer tree based on concatenation of 18 protein markers sequences showing the phylogenetic relationship of 27 *C. kroppenstedtii*-like isolates and the type strain *C. kroppenstedtii* DSM 44385^T^. Branches with bootstrap support of 50% are indicated.

To further confirm the taxonomic identification, the clinical isolates were subjected to whole-genome sequencing. Genomic sequencing results showed that *C. kroppenstedtii*-like group I and group II had a genome size of 2.50 to 2.96 Mb and 2.47 to 2.80 Mb, respectively. The G+C contents of group I and group II were 54.7 to 57.2% and 55.1 to 57.2%, respectively. The genomic relatedness of these isolates and the type strain were calculated by digital DNA–DNA hybridization (dDDH) and average nucleotide identity (ANI) based on the BLASTN algorithm (ANIb). According to the dDDH analyses, 23 isolates (MC-01, MC-04, MC-05, MC-06, MC-08, MC-09, MC-10, MC-11, MC-12, MC-13, MC-15, MC-16, MC-19, MC-20, MC-21, MC-22, MC-23, MC-24, MC-25, MC-26, MC-27, MC-28, MC-29) were observed to belong to the same novel species-level taxon (*C. kroppenstedtii*-like group I), exhibiting 75 to 100% sequence identity to each other and 47.4 to 47.7% to the type strain *C. kroppenstedtii*. Four isolates (MC-02, MC-03, MC-07, MC-17X) were observed to belong to another novel species-level taxon (*C. kroppenstedtii*-like group II), exhibiting 99.6 to 100% sequence identity to each other, 45.5 to 47.8% identity to group I, and 49.9% identity to the type strain. ANIb analysis showed data similar to that of dDDH analysis. *C. kroppenstedtii*-like group I shared 96.99 to 99.99% similarities within themselves and 91.83 to 92.28% similarities to the type strain. *C. kroppenstedtii*-like group II shared 99.92 to 100% similarities between them, 91.83 to 92.28% similarities to group I, and 92.89 to 92.93% similarities to the type strain. Comparative genomic analyses and genomic characteristics of two groups of *C. kroppenstedtii*-like isolates and the closely related type strains are shown in Table 4. The phylogenomic tree based on concatenation of 18 protein marker genes (Fig. 3) indicated that the clinical isolates constituted two clusters separated from *C. kroppenstedtii* DSM 44385^T^, which was consistent with the classification results of dDDH and ANIb.

**TABLE 4:** Comparative genomic analysis and genomic characteristics of two groups of *C. kroppenstedtii*-like isolates and the closest related type strains^*a*^

Strain	Pairwise comparison result^*b*^				Genome characteristic		
	1		2		No. of contigs	Size (Mb)	G+C (%)
	dDDH (%)	ANIb (%)	dDDH (%)	ANIb (%)			
1	97.25 (5.65)	99.59 (0.68)			4–356	2.50–2.96	54.7–57.2
2	45.99 (0.38)	91.97 (0.09)	99.82 (0.15)	99.97 (0.03)	11–108	2.47–2.80	55.1–57.2
3	47.60 (0.06)	92.23 (0.27)	49.90 (0.00)	92.84 (0.04)	1	2.45	57.50
4	21.79 (0.41)	66.81 (0.02)	22.05 (0.17)	66.90 (0.00)	21	2.91	49.8
5	21.43 (0.30)	67.90 (0.11)	24.78 (0.10)	68.16 (0.06)	18	2.59	66.6

a Taxa are indicated as 1, *Corynebacterium parakroppenstedtii* (*C. kroppenstedtii-like* group I); 2, *Corynebacterium pseudokroppenstedtii* (*C. kroppenstedtii*-like group II); 3, Corynebacterium kroppenstedtii DSM 44385^T^; 4, Corynebacterium freiburgense DSM 45254^T^; 5, Corynebacterium timonense 5401744^T^.

b Values are mean with standard deviation.

### Antimicrobial susceptibility.

Based on the CLSI breakpoints for this genus, most isolates of *C. kroppenstedtii*-like group I were sensitive to meropenem, cefepime, vancomycin, daptomycin, gentamicin, and linezolid. Nineteen of the 23 isolates (82%) were resistant to erythromycin and clindamycin, 7 isolates (30%) were resistant to trimethoprim-sulfamethoxazole, 5 (21%) were resistant to ciprofloxacin, and 4 (17%) were resistant to ceftriaxone and tetracycline. The majority of isolates of *C. kroppenstedtii*-like group II were sensitive to meropenem, cefepime, tetracycline, gentamicin, trimethoprim-sulfamethoxazole, linezolid, daptomycin, and vancomycin and resistant to ceftriaxone, ciprofloxacin, erythromycin, and clindamycin. All isolates were intermediate to penicillin. The antimicrobial susceptibility results of the two groups of *C. kroppenstedtii*-like isolates and *C. kroppenstedtii* DSM 44385^T^ are shown in Table 5.

**TABLE 5:** Antimicrobial susceptibilities of the two groups of *C. kroppenstedtii*-like isolates and the type strain

Antimicrobial agent	CLSI breakpoint^*a*^	*C. kroppenstedtii*-like group I MIC (mg/L) (*n* = 23)			*C. kroppenstedtii*-like group II MIC (mg/L) (*n* = 4)				*C. kroppenstedtii* DSM 44385^T^ MIC
		MIC_50_	MIC_90_	Range (% resistant)	MC-02	MC-03	MC-07	MC-17X	
Penicillin	S, ≤0.12; R, ≥4	0.5	2	0.12–2 (0)	2	2	0.5	1	0.12
Ceftriaxone	S, ≤1; R, ≥4	<1	4	<1–4 (17)	4	4	<1	4	2
Cefepime	S, ≤1; R, ≥4	<1	<1	<1 (0)	<1	<1	<1	<1	4
Meropenem	S, ≤0.25; R, ≥1	<0.25	0.5	<0.25–0.5 (0)	<0.25	<0.25	<0.25	<0.25	<0.25
Vancomycin	S, ≤2	<0.5	<0.5	<0.5 (0)	<0.5	<0.5	<0.5	<0.5	<0.5
Daptomycin	S, ≤1	<0.5	<0.5	<0.5 (0)	<0.5	<0.5	<0.5	1	<0.5
Gentamicin	S, ≤4; R, ≥16	<4	<4	<4 (0)	<4	<4	<4	<4	<4
Erythromycin	S, ≤0.5; R, ≥2	>8	>8	<0.25 to >8 (82)	>8	>8	>8	>8	<0.25
Ciprofloxacin	S, ≤1; R, ≥4	<1	4	<1–4 (21)	4	4	4	>8	<1
Tetracycline	S, ≤4; R, ≥16	8	>16	<4 to >16 (17)	<4	<4	>16	<4	<4
Clindamycin	S, ≤0.5; R, ≥4	>4	>4	<0.25 to >4 (82)	>4	>4	>4	>4	<0.25
Trimethoprim-sulfamethoxazole	S, ≤2/38; R, ≥4/76	<0.5/9.5	>4/76	<0.5/9.5 to >4/76 (30)	<0.5/9.5	<0.5/9.5	>4/76	<0.5/9.5	<0.5/9.5
Linezolid	S, ≤2	<1	<1	<1 (0)	<1	<1	<1	<1	<1
Ampicillin	-^*b*^	0.5	2	<0.25 to 2^*b*^	2	2	1	1	<0.25
Levofloxacin	-^*b*^	<2	>8	<2 to >8^*b*^	>8	>8	>8	>8	<2

a S, susceptible; R, resistant.

b -, Non-species-related CLSI breakpoints.

### Detection of resistance genes based on whole-genome sequencing.

Antibiotic resistance genes detected from the genomes of the two groups of *C. kroppenstedtii*-like isolates were not identical. Aminoglycoside resistance gene *APH(3′)-Ia* was detected in 14 isolates of group I (60%) and 2 isolates of group II (50%). Aminoglycoside resistance gene *APH(3′')-Ib* was detected in 18 (78%) and 3 (75%) isolates of *C. kroppenstedtii*-like group I and group II, respectively. Aminoglycoside resistance gene *APH(6)-Id* was detected in 18 group I isolates (78%) and 4 group II isolates (100%). Macrolide, lincosamide, and streptogramin resistance gene *erm*(X) were detected in 17 (74%) and 4 (100%) group I and group II isolates, respectively. Sulfonamide resistance gene *sul1* and tetracycline resistance gene *tet*(W) were detected in 7 (30%) and 17 (74%) *C. kroppenstedtii-*like group I isolates and 1 (25%) and 2 (50%) *C. kroppenstedtii*-like group II isolates, respectively. No antibiotic resistance gene was detected in *C. kroppenstedtii* DSM 44385^T^. The antibiotic resistance genes detection results of two groups of *C. kroppenstedtii*-like isolates and *C. kroppenstedtii* DSM 44385^T^ are shown in Table S1.

## TAXONOMY

### Description of *Corynebacterium parakroppenstedtii* sp. nov.

C*orynebacterium parakroppenstedtii* (pa.ra.krop.pen.stedt’i.i. Gr. pref. para-, besides, alongside, near, like; N. L. gen. masc. n. *kroppenstedtii*, specific epithet of a *Corynebacterium* species; N. L. gen. masc. n. *parakroppenstedtii*, resembling *C. kroppenstedtii*).

The description of the species is based on 23 strains (*C. kroppenstedtii*-like group I). Cells are Gram-positive, lipophilic, rod-shaped, nonmotile, non-spore-forming, catalase-positive, and oxidase-negative. Colonies on Columbia blood agar plates are grayish, smooth, circular, convex, and nonhemolytic with less than 1 mm in diameter after 72 h cultivation at 35°C in the presence of 5% CO_2_ atmosphere. In most strains, acid is produced from glucose but not from sucrose, lactose, xylose, ribose, mannitol, or glycogen. Most strains are positive for pyrazinamidase activity but negative for alkaline phosphatase, β-glucuronidase, β-galactosidase, α-glucosidase, N-acetyl-β-glucosaminidase, and urease activity. Nitrate cannot be reduced. The hydrolysis of gelatin is negative. The hydrolysis of esculin is variable. Most strains are sensitive to meropenem, cefepime, gentamicin, linezolid, daptomycin, and vancomycin but resistant to erythromycin and clindamycin. Intermediate activity to penicillin. Sensitivity to ceftriaxone, ciprofloxacin, tetracycline, and trimethoprim-sulfamethoxazole was strain-dependent.

The type strain is MC-26^T^ (NBRC 115146^T^; CCTCC AB 2020210^T^). It has a DNA G+C content of 56.86%. It was isolated from a breast sample of a patient diagnosed with mastitis in Guangdong Provincial Hospital of Traditional Chinese Medicine in 2019.

### Description of *Corynebacterium pseudokroppenstedtii* sp. nov.

*Corynebacterium pseudokroppenstedtii* (pseu.do.krop.pen.stedt’i.i. Gr. masc./fem. adj. pseudês, false; N. L. gen. masc. n. *kroppenstedtii*, specific epithet of a *Corynebacterium* species; N. L. gen. masc. n. *pseudokroppenstedtii*, a false (*Corynebacterium*) *kroppenstedtii*, resembling *C. kroppenstedtii*).

The description of the species is based on characteristics of 4 strains (*C. kroppenstedtii*-like group II). Cells are Gram-positive, lipophilic, rod-shaped, nonmotile, non-spore-forming, catalase-positive, and oxidase-negative. Colonies on Columbia blood agar plates are grayish, smooth, circular, convex, and nonhemolytic with less than 1 mm in diameter after 72 h cultivation at 35°C in the presence of 5% CO_2_ atmosphere. Acid is produced from glucose but not from sucrose, lactose, xylose, ribose, mannitol, or glycogen. Positive for pyrazinamidase activity but negative for alkaline phosphatase, β-glucuronidase, β-galactosidase, α-glucosidase, N-acetyl-β-glucosaminidase, and urease activity. Nitrate reduction and the gelatin hydrolysis tests are negative. The hydrolysis of esculin is positive. Most strains were sensitive to meropenem, cefepime, tetracycline, gentamicin, trimethoprim-sulfamethoxazole, linezolid, daptomycin, and vancomycin but resistant to ceftriaxone, ciprofloxacin, erythromycin, and clindamycin. Intermediate to penicillin.

The type strain is MC-17X^T^ (NBRC 115143^T^; CCTCC AB 2020199^T^). It has a DNA G+C content of 57.19%. It was isolated from a breast sample of a patient diagnosed with mastitis in Guangdong Provincial Hospital of Traditional Chinese Medicine in 2018.

## DISCUSSION

Available literature and clinical evidence suggest a possible association between *Corynebacterium* infection and mastitis (7, 8, 10, 13). Among the *Corynebacterium* species, *C. kroppenstedtii* was the most common isolate reported in mastitis since its first report published in 2002 (7). Despite increasing data supporting their relationship, the role of *C. kroppenstedtii* in breast pathologies remains unclear, and further studies are urgently required. There was a report that impaired neutrophil responses to Nod2 agonist were associated with granulomatous mastitis due to corynebacteria (16). Recent emerging data also suggest that hyperprolactinemia may be an important risk factor of mastitis caused by *C. kroppenstedtii* (12, 14, 17). Prolactin was thought to modulate the inflammatory response and play a role in mastitis pathogenesis (18). It is noteworthy that 2 patients in our study had hyperprolactinemia and 1 patient had pituitary adenoma, which is a common cause of hyperprolactinemia (19). Most patients in our study received treatment of bromocriptine, a drug for hyperprolactinemia, with better curative effects and outcomes. The exact role of hyperprolactinemia in *C. kroppenstedtii*-related mastitis requires further studies. In our study, all 27 isolates were obtained from breast specimens of female patients with mastitis, supporting the potential pathogenic role of these strains in breast disease and emphasizing that we should pay more attention to the isolation of *Corynebacterium* species in breast specimens.

Isolation is necessary for the successful detection, accurate identification, and antibiotic susceptibility testing of this potential pathogen. The isolation of the lipophilic *Corynebacterium* can be challenging due to the fastidious growth. In our study, the incubation of breast specimens is always at least 72 h at 35°C with 5% CO_2_ to best recover the fastidious bacteria, which is also applicable to other clinical laboratories. On account of the lipophilic nature, the addition of a lipid component, such as Tween80, is often used to improve the culture yield. A medium that contains galactose, Tween 80, and fosfomycin has been specifically designed for the isolation of *C. kroppenstedtii* (20). The accurate identification of *Corynebacterium* species has become more reliable with the availability of MALDI-TOF MS and molecular techniques in the clinical laboratory (21). MALDI-TOF MS is a powerful tool to identify organisms to both genus and species levels rapidly and accurately and has been widely used in clinical laboratories. API kit assay is another method for rapid identification of fastidious bacteria, but it is not always suitable for species like *C. kroppenstedtii*. The API Coryne (bioMérieux, France) was designed in the early 1990s, but its databases have been updated only infrequently. Currently, gene sequencing is still the gold standard for microbial identification. In the present study, the use of phenotypic characterization can hardly identify and differentiate the two groups of *C. kroppenstedtii*-like isolates and the type strain. MALDI-TOF MS using the Vitek MS system and Bruker Biotyper system also failed to distinguish these isolates from *C. kroppenstedtii*. Based on the dendrogram produced by the Bruker Biotyper system, only *C. kroppenstedtii*-like group I was separated from the type strain. The partial 16S rRNA gene identity (99.6 to 99.9%) also could not distinguish these isolates from *C. kroppenstedtii*. These isolates, however, showed 96.2 to 98.1% partial *rpoB* gene and 97.3 to 97.7% partial *fusA* gene similarity to *C. kroppenstedtii*, demonstrating that *rpoB* and *fusA* genes sequencing allow more accurate identification for *C. kroppenstedtii*-like strains, as they are significantly more polymorphic than the 16S rRNA gene. Previous reports have shown that *rpoB* gene sequencing is a better option to identify and differentiate *Corynebacterium* species (22, 23). In this study, whole-genome sequencing was found to be the ultimate tool to distinguish and identify two new species belonging to the genus *Corynebacterium*.

There is no consensus for optimal management of *Corynebacterium* breast infection with treatment options such as surgical excision, corticosteroids, or antibiotics treatment. However, some reports showed that antibiotics play a marginal role in the natural treatment of this disease (7, 10, 24). Most patients in our study received traditional Chinese medicine, bromocriptine, and surgical debridement treatment. The traditional Chinese medicine decoction used for treatment is Kuijian Xiaoju Tang, which is composed of mainly *Radix Bupleuri*, *Fructus Tribuli*, *Smilacis Glabrae Rhizoma*, *Gleditsiae Spina*, *Angelicae Dahuricae Radix*, *Radix Trichosanthis*, *Angelicae Sinensis Radix*, *Paeoniae Radix Rubra*, *Radix Rhizoma Glycyrrhizae*, and *Prunellae Spica*. A small number of patients also received dexamethasone and antibiotic treatment like rifampicin, isoniazide, and ethambutol. Most patients had a good outcome after treatment. There is also recent clinical research suggesting that traditional Chinese medicine is effective in treating mastitis, indicating the potential advantage of traditional Chinese medicine (25, 26).

For *C. kroppenstedtii* breast infection, the data about antimicrobial treatment options are limited, and some studies have reported that *C. kroppenstedtii* isolates are susceptible to most antibiotics except for fosfomycin using the disk diffusion method or the E test (24, 27–29). Meanwhile, resistance to penicillin (10, 11), imipenem (14), erythromycin (12, 30), trimethoprim-sulfamethoxazole (28, 30), and clindamycin (10, 12, 30) has been reported. In this study, most *C. kroppenstedtii*-like isolates were susceptible to meropenem, cefepime, vancomycin, daptomycin, gentamicin, and linezolid. Isolates’ resistance to ceftriaxone, ciprofloxacin, erythromycin, tetracycline, clindamycin, and trimethoprim-sulfamethoxazole was found. There is a possibility of a difference in antibiotic susceptibility with different genospecies. Accordingly, correct species identification and antimicrobial susceptibility testing would ideally be performed for all isolates.

The resistance genes detected using the genomes of 27 clinical isolates included *APH(3′)-Ia*, *APH(3′')-Ib*, *APH(6)-Id*, *erm*(X), *sul1*, and *tet*(W), which are known from other corynebacteria (31–33). Comparing the prediction of antibiotic resistance genes with the results of *in vitro* susceptibility testing, the results can be summarized to the following points. (i) Twenty-three isolates were resistant to both erythromycin and clindamycin, while 21 isolates were found to have the corresponding antibiotic resistance gene *erm*(X). (ii) Tetracycline and sulfonamide antibiotic resistance genes were detected in 19 isolates and 8 isolates, respectively, but their susceptibility testing of tetracycline and trimethoprim-sulfamethoxazole was variable. (iii) Aminoglycoside resistance genes were detected in 22 isolates, but all of them were sensitive to gentamicin. (iv) None of the isolates were found to have β-lactam and quinolone resistance genes, but the response of all isolates to penicillin was intermediate; some isolates were intermediate or resistant to ceftriaxone, meropenem, and ciprofloxacin. By combining the *in vitro* susceptibility testing and the prediction of antibiotic resistance genes, we may be able to prioritize treatment with antibiotics other than macrolide, lincosamide, and tetracycline. These results indicate that not only are the antibiotic resistance and antibiotic susceptibility profiles of the same genospecies different, but the prediction of the antibiotic resistance gene profile is also different from the actual antibiotic resistance. This phenomenon may be related to differences in the expression of resistance genes in different strains. Antibiotic resistance genes exist not only in chromosomes but also in plasmids. Some isolates showing antibiotic resistance by *in vitro* susceptibility testing with no corresponding antibiotic resistance genes being detected may be due to the incompleteness of the draft genome. In addition, the inconsistencies between the antibiotic susceptibility of some isolates and the detected antibiotic resistance genes may be attributed to the low survival pressure of *in vitro* culture and the variation after serial passages. All these mentioned resistance genes are predicted for reference. The actual existence and expression of resistance genes need to be further verified by molecular methods. For clinical treatment, it may be feasible to classify through large amounts of actual antibiotic susceptibility data.

In conclusion, the *C. kroppenstedtii*-like clinical isolates associated with mastitis in our study represent two novel genospecies within the genus *Corynebacterium*, for which the names *Corynebacterium parakroppenstedtii* sp. nov. (*C. kroppenstedtii*-like group I) and *Corynebacterium pseudokroppenstedtii* sp. nov. (*C. kroppenstedtii*-like group II) are proposed. Our work is an important documentation of the identification of important potential pathogens for mastitis and provides hints that we should pay more attention to the isolation of *Corynebacterium* species in breast specimens. Our work also provides the antibiotic susceptibility profiles and suitable identification methods for these two novel species. By sharing the descriptions of two novel species as well as our experience in the identification, we hope that further epidemiological investigation of these strains can be performed and that their role in mastitis can be explored.

## MATERIALS AND METHODS

### Strains.

Twenty-seven *C. kroppenstedtii*-like isolates were isolated from clinical breast specimens in Guangdong Provincial Hospital of Traditional Chinese Medicine from 2017 to 2019, and the strain *C. kroppenstedtii* DSM 44385^T^ was included in the present study as a reference type strain. Basic information of these *C. kroppenstedtii*-like isolates was outlined in Table 1. The purified isolates were routinely maintained by subculturing on Columbia blood agar plates at 35°C in a humidified atmosphere supplemented with 5% CO_2_ and stored as glycerol suspensions (30%, vol/vol) with 2% blood at −80°C.

### Phenotypic testing and MALDI-TOF MS.

These isolates were initially identified by phenotypic characteristics and MALDI-TOF MS. Microscopic characteristics were determined by Gram stain. Lipid requirement was tested by comparing cultures grown on brain heart infusion broth and Columbia blood agar with cultures grown on these media supplemented with 1% Tween 80 after 3 days at 35°C with 5% CO_2_. Biochemical characterizations were performed using commercial API Coryne (bioMérieux, France) according to the manufacturer’s instructions. API Web was used to interpret the API codes. MALDI-TOF MS was carried out by both Bruker Biotyper system (BDAL Library; Bruker Daltonics, Germany) and Vitek MS system (V2.0; bioMérieux, France) according to the manufacturer’s instructions. The percentage similarities of identical mass peaks obtained by the Bruker Biotyper system (Bruker Daltonics, Germany) were calculated and used to generate dendrogram by the statistical toolbox of Matlab 7.1 (MathWorks Inc., USA) integrated into the MALDI Biotyper 2.0 software.

### DNA sequencing and analysis.

Genomic DNAs were extracted using a bacterial genomic DNA extraction kit (AG, China) according to the manufacturer’s instructions. All clinical isolates were subjected to partial sequencing of the 16S rRNA, *rpoB*, and *fusA* genes. The protocols and primers of PCR amplification and Sanger sequencing were performed as described previously (22, 34, 35). The sequences were assembled using DNAMAN (version 7) software and compared with those related type strains on the NCBI BLAST website (https://blast.ncbi.nlm.nih.gov/Blast.cgi). Phylogenetic trees were constructed using the neighbor-joining method with 1,000 bootstrap replications in the MEGA (version 7) software (36).

All isolates were subjected to whole-genome sequencing for obtaining a clear species differentiation. Genomic DNAs were extracted using a bacterial genomic DNA extraction kit (AG, China) and sequenced using Illumina NovaSeq PE150. The cleaned data were then assembled using SPAdes version 3.14.0. (37). The assembly was integrated with CISA software (38). The least scaffolds were selected to obtain the draft genome. For the construction of the phylogenomic tree, the marker genes were retrieved from the draft genomes of the 27 clinical isolates and *C. kroppenstedtii* DSM 44385^T^ using AMPHORA2 (39). The 18 corresponding marker genes were listed as follows: *frr*, *infC*, *nusA*, *pyrG*, *rplA*, *rplC*, *rplD*, *rplE*, *rplK*, *rplM*, *rplT*, *rpmA*, *rpsE*, *rpsJ*, *rpsK*, *rpsM*, *smpB*, *tsf*. The sequences were aligned separately using MUSCLE (40) and concatenated by using a Perl script (https://github.com/nylander/catfasta2phyml). The protein-concatemer tree was established by comparing concatenated amino acids using the RAXML method (41). The tree was visualized through the online Tree of Life program version 6.5 (42). Genomic relatedness of these clinical isolates with the type strain was estimated using digital DNA–DNA hybridization (dDDH) and average nucleotide identity (ANI) based on the BLASTN algorithm (ANIb). dDDH values were calculated using the recommended settings (formula 2) of the Genome-to-Genome Distance Calculator 2.1 (43). ANIb values were calculated using JSpeciesWS (http://jspecies.ribohost.com/jspeciesws/).

### Antimicrobial susceptibility testing.

Antibiotic susceptibility testing was carried out with the broth microdilution method by use of *Corynebacterium* ID&AST kit (TDR, China) according to the manufacturer’s instructions. The CLSI standard for determination and interpretation of antimicrobial MICs for *Corynebacterium* spp. was applied for the following antibiotics: penicillin, ceftriaxone, cefepime, meropenem, vancomycin, gentamicin, erythromycin, daptomycin, tetracycline, trimethoprim-sulfamethoxazole, ciprofloxacin, clindamycin, and linezolid (44).

### Prediction of antibiotic resistance genes from the whole-genome sequencing data.

The Comprehensive Antibiotic Resistance Database (CARD; https://card.mcmaster.ca) (45) was used to predict antibiotic resistance genes in the whole-genome sequences. Resistance Gene Identifier (RGI 5.2.0, CARD 3.1.3) was used with open reading frame (ORF) prediction using Prodigal, homolog detection using DIAMOND, and strict significance based on CARD curated bitscore cutoffs. The “Perfect” and “Strict” default settings for sequence analysis were chosen as selection criteria.

### Data availability.

The 16S rRNA, *rpoB*, and *fusA* gene sequences of the clinical isolates were deposited in GenBank with accession numbers MW819649 to MW819675 and MZ031080 to MZ031133 (Table 2). The Whole Genome Shotgun projects of the clinical isolates have been deposited at DDBJ/ENA/GenBank under the accession numbers JAFFSY000000000, JAEUWU000000000, JAGSNZ000000000, JAGSOA000000000, JAGSNY000000000, JAKKFA000000000, JAKJKO000000000 to JAKJLB000000000, JAKKNX000000000 to JAKKOA000000000, and JAKLTI000000000 to JAKLTK000000000 (Table 2).
