# Supplementary material for: Classification of 27 Corynebacterium kroppenstedtii-Like Isolates Associated with Mastitis in China and Descriptions of C. parakroppenstedtii sp. nov. and C. pseudokroppenstedtii sp. nov
Source: Microbiol Spectr. 2022 Mar 15;10(2):e01372-21. doi: 10.1128/spectrum.01372-21 (PMC9045094; doi:10.1128/spectrum.01372-21)
Supplement: SUPPLEMENTAL FILE 1 — Supplemental material. Download SPECTRUM01372-21_Supp_1_seq4.pdf, PDF file, 0.1 MB [file spectrum01372-21_supp_1_seq4.pdf]

Classification of 27 *Corynebacterium kroppenstedtii*-like Isolates Associated with Mastitis in China and Descriptions of *C. parakroppenstedtii* sp. nov. and *C. pseudokroppenstedtii* sp. nov.

Qiang Luo,[a,b] Qianming Chen,[b] Junhui Feng,[b] Tianqi Zhang,[b] Li Luo,[b] Cha Chen,[a,b] Xiaoyan Liu,[c] Ning Xu,[a,b] Pinghua Qu[a,b]

Department of Clinical Laboratory, The Second Affiliated Hospital of Guangzhou University of Chinese Medicine, Guangdong Provincial Hospital of Traditional Chinese Medicine, Guangzhou, China[a]; The Second Clinical College, Guangzhou University of Chinese Medicine, Guangzhou, China[b]; Department of Breast Centre, The Second Affiliated Hospital of Guangzhou University of Chinese Medicine, Guangdong Provincial Hospital of Traditional Chinese Medicine, Guangzhou, China[c].

Address correspondence to Pinghua Qu, ququtdr@163.com, or Ning Xu, xu_ning21@163.com

Q.L. and Q.C. contributed equally to this article.

Running Title: Two Novel *Corynebacterium* Species Related to Mastitis

TABLE S1 Detection of antibiotic resistance genes in two groups of *C. kroppenstedtii*-like isolates and the type strain

Strain no.	Antibiotic resistance genes[a]						Total
	Aminoglycoside resistance genes *APH(3')-Ia*	Aminoglycoside resistance genes *APH(3'')-Ib*	Aminoglycoside resistance genes *APH(6)-Id*	Macrolide, lincosamide and streptogramin resistance gene *erm(X)*	Sulfonamide resistance gene *sul1*	Tetracycline resistance gene *tet(W)*	
TS[b]	–	–	–	–	–	–	0
***C. kroppenstedtii*-like group Ⅰ**							
MC-01	–	–	–	–	–	–	0
MC-04	+	+	+	+	–	+	5
MC-05	+	+	+	–	+	+	5
MC-06	+	+	+	–	+	+	5
MC-08	–	+	+	+	–	+	4
MC-09	+	+	+	+	–	+	5
MC-10	–	+	+	+	–	+	4
MC-11	+	+	+	+	+	+	6
MC-12	+	+	+	+	–	+	5
MC-13	+	+	+	+	–	+	5
MC-15	–	+	+	+	–	+	4
MC-16	+	+	+	+	–	+	5
MC-19	+	+	+	+	+	+	6

MC-20	+	+	+	+	+	+	6
MC-21	+	+	+	+	+	–	5
MC-22	+	+	+	+	–	+	5
MC-23	+	+	+	+	+	–	5
MC-24	–	–	–	–	–	–	0
MC-25	–	+	+	+	–	+	4
MC-26	–	–	–	–	–	–	0
MC-27	+	+	+	+	–	+	5
MC-28	–	–	–	+	–	+	2
MC-29	–	–	–	–	–	–	0
Total	14	18	18	17	7	17	91
***C. kroppenstedtii*-like group Ⅱ**							
MC-02	–	+	+	+	–	–	3
MC-03	–	+	+	+	–	–	3
MC-07	+	–	+	+	+	+	5
MC-17X	+	+	+	+	–	+	5
Total	2	3	4	4	1	2	16

[a] –, not detected; +, detected.

[b] TS, the type strain *C. kroppenstedtii* DSM 44385^{T}.
